# Factors to consider during the implementation of nutrition and physical activity trials for people with psychotic illness into an Australian community setting

**DOI:** 10.1186/s12913-020-05629-0

**Published:** 2020-08-12

**Authors:** Doreen Mucheru, Samantha Ashby, Mary-Claire Hanlon, Mark McEvoy, Lesley MacDonald-Wicks

**Affiliations:** 1 Faculty Health and Medicine, The University of Newcastle, Callaghan, 2308 Australia; 2 Priority Research Centre for Brain and Mental Health, The University of Newcastle, Callaghan, 2308 Australia; 3 Brain and Mental Health Program, Hunter Medical Research Institute, New Lambton, 2305 Australia; 4 Hunter Cancer Research Alliance, The University of Newcastle, Callaghan, 2308 Australia; 5 Centre for Clinical Epidemiology & Biostatistics, Hunter Medical Research Institute, New Lambton, 2305 Australia; 6 Priority Research Centre for Physical Activity and Nutrition, The University of Newcastle, Callaghan, 2308 Australia

**Keywords:** Lifestyle interventions, Psychotic illness, Mental health, Knowledge translation, Community managed organisations

## Abstract

**Background:**

Research in lifestyle interventions focusing on nutrition and physical activity in people living with psychotic illness, highlights anthropometric and metabolic benefits of these interventions. However, little is known about potential factors to consider during implementation into real-world contexts. Community-managed organisations (CMOs) that provide services for people with mental illness, offer an ideal implementation context for lifestyle interventions. Successful translation of lifestyle interventions into CMOs may be achieved though considering the factors associated with program access and delivery in these settings. This study primarily aimed to identify the factors that affect program access in a local CMO from the perspective of consumers and staff. The secondary aim was to describe the elements that impact on program delivery from the perspective of staff.

**Methods:**

Thirteen semi-structured interviews were conducted with 6 consumers and 7 staff in a CMO in regional Australia. Topics explored in interviews were based on implementation concepts identified in the “Integrated Promoting Action on Research Implementation in Health Systems” (i-PARIHS) knowledge translation framework. Thematic data analysis was conducted using Nvivo software.

**Results:**

Emergent themes on issues that influenced program access were (1) consumer financial status, domestic responsibilities, and health; (2) the design and delivery of programs; (3) structure and practices of the organisation; (4) attitude, skills and effort of staff involved in program delivery; and (5) social connections and stigma experienced by consumers during program access. Moreover, staff perceptions on elements that impacted program delivery highlighted themes on consumer attendance and interest in prospective programs, availability and restrictions to the use of funding, as well as the organisational structure and practices.

**Conclusions:**

The factors affecting program access and delivery can generally be managed or planned for during the design of lifestyle interventions and subsequent translation into the CMO context. However, resolution of issues related to consumer financial status and health requires the collaboration of various government sectors for system-wide solutions.

## Background

Psychosis is a condition marked by the experience of delusions, hallucinations, psychomotor retardation and catatonic behaviour (apparent unresponsiveness to external stimuli) [1]. The experience of psychosis is predominant in people with schizophrenia spectrum disorders, or in those with mood or affective disorders but can also occur in the absence of a specific mental health diagnosis [1]. Psychotic disorders are categorised as severe or serious mental illness due to the intensity of symptoms that occur, and the extent of disability that ensues [2]. People with psychotic illness and those with other severe or serious mental illness often present with similar socioeconomic, cognitive, clinical and functioning profiles; thus research pertaining to the physical health of these groups is often generalizable [3].

Worldwide research shows that people with psychotic illness have a 53% (odds ratio [OR] 1.53; 95% CI 1.27–1.83) higher risk of having cardiovascular disease compared to a regionally matched sample from the general population [4]. This is primarily due to the combined effects of lifestyle risk factors which include high rates of smoking, poor dietary choices and inadequate physical activity [4]. Research on the management of these lifestyle risks is a current focal point; results show that lifestyle interventions, which focus on nutrition and physical activity among people with psychotic illness, produce significant efficacy [5]. Meta-analytic comparisons of randomised controlled trials (RCTs) for these interventions, display mean improvements in weight (− 4.1 kg; 95% CI -7.77, − 2.76, *p* < 0.000), body mass index ([BMI] -2.9 points; 95% CI -1.78, − 0.36, *p* = 0.003), waist circumference (− 2.2 cm; 95% CI -6.9, − 0.46, *p* = 0.025), total cholesterol (− 20.98 mg/dL; 95% CI -33.78, − 8.19, *p* = 0.001), triglycerides (− 61.68 mg/dL; 95% CI -92.77, − 30.59, *p* = 0.0001) and fasting blood glucose (− 5.79 mg/dL; 95% CI-9.73, − 1.86, *p* = 0.004), when compared to control conditions [5, 6]. Despite established efficacy, evidence-based lifestyle interventions in Australians with psychotic illness are primarily conducted for research, then discontinued after the trial period [6–8]. Moreover, a systematic review on the possible incentives and barriers of participating in lifestyle interventions among people with severe mental illness (generally) did not identify any relevant publications [9]. Existing lifestyle interventions however noted that research participation is influenced by illness symptoms, medication effects, transport provision, financial challenges, staff attributes, intervention characteristics, stigma and inclusion of staff in interventions [9]. Successful implementation of lifestyle interventions for people with psychotic illness requires broader information on the factors that could influence external validity of research [7]. Furthermore, knowledge is limited to existing trials because longitudinal work is lacking [5–7].

Existing lifestyle intervention research not only focuses on quantifying impact but also identifying efficacious strategies [5–8]. A recent network meta-analysis shows that combined use of education, personalised goals or plans, and progress review in the dietary and physical activity intervention components, lead to the greatest mean decreases in weight (− 4.1 kg) and BMI (− 2.9 points) [5]. Nonetheless, evidence on implementation of these RCTs following research is unavailable and expert groups have identified the need for implementation research in this field [5–8, 10, 11]. Knowledge translation frameworks can guide implementation research by identifying factors that require consideration when translating evidence into practice [12]. These frameworks recognise that successful implementation requires consideration of priorities at the proposed setting; the feelings, attitudes, motivation, goals, knowledge, skills and resources of those who influence or receive the proposed change; and the role of the internal and external context in the knowledge translation process [12]. Investigation of these considerations, as they relate to the implementation of lifestyle interventions for people with psychotic illness, could provide rich detail on features that promote external validity [7, 12].

Community managed organisations (CMOs) offer feasible knowledge translation contexts for lifestyle interventions, because they provide community-based programs for Australians experiencing psychotic illness [13]. Previously known as the non-government sector, CMOs complement the efforts of the public mental health or government services, in community-based program delivery for people with psychotic illness [14]. Community-based support gained prominence following deinstitutionalisation of Australian mental health care, shifting treatment from long-stay psychiatric hospitals to community contexts [15]. Programs available through both the CMO and public mental health system aim to facilitate independence, minimise disability and promote overall recovery [14]. Australians with psychotic illness are, however, more likely to utilise programs in the CMO sector (22.4%) than those in the government sector (14.5%), which makes CMOs a more desirable implementation setting [14]. Additionally, CMOs offer psychosocial support, along with other services that target prevention, rehabilitation and early intervention for mental illness [13, 16]. Psychosocial services promote community engagement though employment, health promotion, social interaction and housing [13, 16].

CMOs were primarily funded by the Commonwealth or State Governments, with supplementary contributions made by philanthropic trusts and foundations [17, 18]. In 2013, the funding of disability support services (including psychiatric disability) commenced a transition to the National Disability Insurance Scheme (NDIS), which provides individualised plans and funding through the National Disability Insurance Agency (NDIA) [19, 20]. The shift presents various concerns for consumers with psychiatric disability including underestimation of persons eligible; flexibility of the scheme to support fluctuating needs of consumers; and boundaries of services accessed via the NDIS versus the rest of the health system [19]. Despite this, NDIS consumers now purchase services directly from CMOs, which gives them control over the funding [19, 20].

Identifying the various factors that affect program access and delivery in the CMO setting, allows for consideration of these issues during translation of evidence-based lifestyle interventions which focus on nutrition and physical activity [12]. The aim of this study was to identify the factors that affect program access in a local CMO from the perspectives of both consumers and staff. Secondary to this, was to describe the elements that impact on program delivery from the perspective of staff.

## Methods

### Study design

This is an original study that was designed by the research team. The “Integrated Promoting Action on Research Implementation in Health Systems” (i-PARIHS) knowledge translation framework aided the identification of suitable participant groups, and the construction of semi-structured interview questions that were pertinent to evidence-based translation of programs (interview guides provided as additional file 1) [12]. The i-PARiHS framework highlights the importance of identifying local circumstances and priorities during evidence-based knowledge translation [12]. Additionally, the feelings, attitudes, motivation, goals, knowledge, skills and resources of those who influence or are affected by the implementation, ought to be sought [12]. Finally, contextual information on the local and external implementation setting supports evidence-based knowledge translation [12]. These implementation concepts were also used in the development of study aims [12].

Topics explored in consumer interviews focused on the programs already accessed at the CMO, and enablers and barriers that affected their attendance. In contrast, interviews for staff involved in program delivery assessed how the programs were developed, factors that affected consumer attendance of programs, and considerations prior to the introduction of new programs. Moreover, interviews for staff who oversaw program delivery delved into how existing programs were initially developed, selected, evaluated, and introduced into the setting. These topics endeavoured to provide an appreciation of the circumstances at the CMO, considerations that influenced program access, and contextual factors likely to impact on program delivery.

### Setting and participant recruitment

This study was conducted at a CMO that provides programs and services to people with severe mental illness, in a regional city in New South Wales, Australia. Approximately 25 consumers were accessing programs at the organisation in 2019. Programs focused on improving different aspects of health and wellbeing, and were run for about 2–4 h each day.

Initial permission to conduct the study was obtained by MCH from the CMO manager. MCH and DM provided the CMO manager with full details of the project and the purpose of the research. DM (female PhD candidate) then sought written and informed consent to recruit from the CMO. DM volunteered at the CMO to gain familiarity with the context, form rapport with staff and consumers, and determine suitable recruitment methods. Staff and consumers at the CMO were aware of the intent to carry out research in the setting. Inclusion criteria for staff included direct involvement with delivery of consumer programs, or overseeing delivery of consumer programs. Staff were verbally invited into the study by DM. Staff who expressed interest were provided with a study information statement.

Consumers who were interested in the study were referred by staff. Referrals were made if participants were 18 years and over, had access to CMO programs, and could consent to and participate in interviews with minimal risk of distress. Staff requested eligible and willing consumers to fill out a consent form, approving phone or email contact from DM for further study information. Those expressing interest were provided with a study information statement.

### Data collection and consent

Interviews were completed following written and informed consent, when convenient to participants, and conducted on CMO premises by DM (interview guides provided as additional file 1). DM was mentored on the process of carrying out interviews by MCH who has previously coordinated large scale studies (*n* > 200) that utilise this mode of data collection. MCH has been trained in the delivery of structured and semi-structured interviews which focus specifically on psychotic disorders, for example the Diagnostic Interview for psychoses (DIP), and in the administration of cognitive assessments such as Wechsler Adult Intelligence Scale (WAIS) [21, 22]. One-on-one, semi-structured interviews were conducted, with none of the interviews repeated. Field notes were generally made after the completion of interviews. All interviews were audio-recorded, and the interview durations ranged between 15 and 40 min. As part of the member-checking process, participants were provided the option of reviewing interview transcripts. Researchers did not anticipate data saturation would be attained during the current study, due to the small number (*n* = 25) of people the CMO serviced during the research period.

Six consumer interviews were completed, with all those referred agreeing to participate. Five staff involved in program delivery took part in interviews, and this figure excluded two staff who did not show an interest in participating. Two CMO managers were interviewed, based on their oversight of program delivery. A transition in CMO leadership during the research period allowed for two interviews instead of one.

### Analysis

Interviews were transcribed verbatim. Transcripts were assessed for accuracy by DM via comparison with audio data. Thematic data analysis as prescribed by J Saldaña [23] was completed by DM using NVivo12—a computer assisted data analysis package. Data were coded and similar codes clustered into themes and subthemes. Support for this process was provided by SA.

## Results

### Sample characteristics

Thirteen participants took part in the present study: six were consumers experiencing a severe mental illness and seven were staff at the CMO. Of the seven staff, five were support workers and two were managers.

### Staff and consumer perceptions of the factors that affect program access

Perceptions of the factors that affect program access yielded five themes, which are discussed in subsequent sections (refer to Table 1 for summary information).

**Table 1:** Staff and consumer perceptions of the factors that affect program access

*Theme*	Short Description	Subtheme
*1.1 Consumer financial status, domestic responsibilities and health*	The various aspects of consumers’ personal lives that impacted on program access	Health status
		Medication side effects
		NDIS financial support for programs and additional program costs that are non-reimbursable by NDIS
		Domestic responsibilities
		Motivation
*1.2 Program design and delivery*	The different aspects of programs that affected attendance	Activities offered
		Location convenience
		Transport provision
		Time of day programs are conducted
		Annual timing of programs
		Consideration of individual consumer needs
		Perceptions about programs
*1.3 Organisational structure and practices*	Determinants of program access that relate to the CMO and how it was run	Lack of equipment
		Dissemination of program information
*1.4* S*taff attitude, skills and effort*	The effect of staff involved with program delivery on attendance	Attitude and professional skills
		Provision of follow-up
*1.5 Social connections and stigma*	The effects of social factors on program access	Social connections with those in program
		Stigma associated with mental illness

### Consumer financial status, domestic responsibilities and health

This theme describes the various aspects of consumers’ personal lives that impacted program access.

An issue raised by both groups was the impact of consumers’ health status on program attendance. Poor health was a consequence of physical or mental illness, and was often a deciding factor for program attendance. Staff 4 stated:

*“The thing is, often they might get sick. Or they don't come in one week, and then that just sets ... They won't come in the next week, and then the next week. Or some of the clients have had to go to hospital, cause they'll have a lapse* [*sic*]*.”*

In addition, consumers and staff participants recognised that medication side effects contributed to fatigue, which limited the capacity to attend programs. Consumer 4 summarised this:

*“Because I take medication at night, in the morning I'm pretty groggy and it's hard to wake up, and hard to get going. That's probably what it is, mainly.”*

Commencement and continuation with programs was limited by lack of NDIS financial support and by additional program costs that were non-reimbursable under the NDIS, as reported by consumers and staff. Additionally, consumers were often on a budget as illustrated by Consumer 1:

*“I'm paying a lot of things, I'm only on the pension so I only have a couple of days of work a week so, it all adds up.”*

Consumers, but not staff, reported that domestic responsibilities and chores were an important overall determinant of attendance. Consumer 5 expressed how family responsibilities affected her capacity to attend programs:

*“And apart from the children's problems, my husband has a terminal illness. So, if he is not well, or if he needs to go to the hospital, or stuff like that, that will stop me from attending groups as well.”*

One staff participant added that consumer motivation could positively, or negatively, affect program attendance. Staff 4 described this in the statement:

*“But the ones that have come, they've wanted to come. But they haven't had the transport, and no* [*sic*] *support worker.”*

However, consumer and staff reports indicated that consumer motivation could be altered through positive encouragement from staff. Staff 5 described this by stating:

*“So sometimes, it's really pushing them out of their comfort zone. But I say, I always encourage. I say, ‘It's just a little step. It's a little step. Think about the future. It's another step.”*

### Program design and delivery

This theme related to the different aspects of programs that affected attendance.

Consumers and staff agreed that activities offered in programs influenced whether consumers enjoyed the programs, which affected continued program attendance. Consumer 2’s response to why she continued to attend one of the available programs was:

*“I like doing things with the people in the group and* [states name] *makes up interesting things for us to do each month, so that's really good.”*

Inconvenient program location was cited as a potential barrier to attending existing programs by consumers and staff. Convenience was based on proximity of program activities to consumer residence (as programs were not always within CMO premises), or difficulties associated with accessing program meeting spaces within the CMO. Staff 4 highlighted this in the comment:

*“Some people find it daunting coming in here, because it's up the stairs. It's a physical barrier to the space itself.”*

Providing consumers with transport to the venue was described by consumers and staff as a way of facilitating program attendance because consumers experienced transport difficulties associated with the inability to drive, or catch public transport. Staff 2 reported:

*“All of our clients don't travel [*sic*], so travel training and being able to pick them up and things, so unless they can get into centre, a lot of them can't get here and a lot of them just won't take public transport.”*

Consumers and staff stated that the timing of programs affected the consumer’s capacity to attend. This was primarily due to medication side effects (which included feelings of lethargy early in the morning). Staff 7 summarised this by stating:

*“We talked to the guys, they don't like groups early morning. They all struggle to get up and get motivated, and out the door, and that's a lot around their* [*sic*] *medication that they take.”*

One staff participant mentioned that seasonal timing of programs affected attendance because interruption of programs by holidays resulted in consumers failing to return. This is how Staff 4 explained her experience regarding consumers’ initial response to a program:

*“Initially very good. We had about six people, which I think is quite big for here. They thought it was really good. Just ... … ... I think there was a holiday that happened.”*

Staff added the importance of considering individual consumer needs when planning program sessions, and involving consumers in planning and executing programs. This provided a sense of ownership, and contributed to enjoyment, participation in program activities, and attendance. Staff 6 expressed this in a response on consumer incentives for attending existing programs:

*“If they feel that their voices is* [*sic*] *being heard and if they feel that they're going to get something out of it—everybody would be different … … … . You know, so it's gonna* [*sic*] *be different for everyone who comes and I think what you gotta* [*sic*] *do, you’ve got to be flexible to be able to cater for everybody's needs.”*

Finally, two staff participants noted the importance of ensuring consumers had the right perceptions about what programs entailed, to prevent anxiety pertaining to the unknown which could influence their attendance. When asked why consumers cancelled participation in certain programs, Staff 5 said:

*“Some of it is to do with their perception of what it's going to be.”*

### Organisational structure and practices

This theme comprised determinants of program access that relate to the CMO and how it was run.

One consumer reported that a lack of equipment for those with mobility disabilities hindered program attendance; however, this was later rectified. Consumer 2 reported this by saying:

*“I couldn't come for such a long time to things because they're all up here and they didn't have a chair lift … … .. And there are other people who are older, who won't use the stairs either because they're very steep.”*

Staff indicated that dissemination of program information to CMO consumers was poor as it was not always clear whose responsibility it was, or how it was to be done. When asked whose responsibility it was to distribute fliers with information on consumer programs, Staff 2 stated:

*“That's the problem. Not every support worker comes into the office and not every support worker would print them at home and hand them to their clients”.*

### Staff attitude, skills and effort

This theme described the effect of staff involved with program delivery on attendance.

Consumers and staff admitted that the attitude and professional skills of staff affected rapport with consumers, which was a motivator for continuing with programs. Important staff characteristics included friendliness, empathy, showing interest in the lives of consumers, providing step-by-step guidance, and well-planned program sessions. Consumer 5 described the effect staff can have in her decision to attend programs by explaining:

*“Depending on the support worker that I have, ‘cause, I get along with some better than others. Some of them, if they came to my house and talked to me for a while, they can probably twist my arm to come.”*

In addition, follow-up provision was regarded as essential by consumers and staff. Follow-up included checking why consumers could not attend program sessions, or providing attendance reminders. Consumer 4 commented:

*“I think I get enough support. Like* [states name] *rings me and says, each second Wednesday morning, ‘Am I coming?’”*

### Social connections and stigma

The effect of social factors on program access was presented in this theme.

Attaining strong social connections with those attending programs motivated program attendance as per consumer and staff reports; one manager recognized that staff could facilitate and foster a social environment during program delivery. Consumer 6 described his objective for attending one of the available programs:

*“I was very, very shy and very shell-shocked for the first 10 years of the group. It's only in the last couple of years that I've started to spread my wings, you might say. So, people will remember me as being particularly shy. But I get social connections out of it.”*

In contrast, stigma associated with mental illness was considered a possible deterrent of attendance by staff. They perceived that consumers had a fear of being recognised as having a mental illness or associating with those with mental illness. When asked why some consumers failed to attend programs, Staff 3 responded:

*“Some don't want to identify with other people with mental health problems and we only have mental health clients.”*

Furthermore, one consumer expressed satisfaction with a service delivery name change that no longer reflected a focus on mental illness, due to the associated stigma. Consumer 2 stated:

*“Every time somebody gets killed or something happens, it's always someone with schizophrenia. So, I was really glad when* [states the service name] *changed its name.”*

### Staff perceptions on the elements that impact on program delivery

Three themes emerged from staff perceptions on elements that impacted on program delivery, that is introducing or running programs (refer to Table 2 for summary information).

**Table 2:** Staff perceptions on the elements that impact on program delivery

*Themes*	*Short Description*	*Subthemes*
*2.1 Consumer attendance and interest*	Consumer-related factors that affected program delivery	Attendance
		Interest in prospective programs
*2.2 Availability and restrictions to the use of funding*	The availability of funding or the stipulated uses of financial resources	Cost-effectiveness of programs
		Lack of funding
		Restrictions on how funding can be used
*2.3 Organisational structure and practices*	Matters pertaining to the CMO and how it was run	Facility availability
		Policies and procedures
		Staff input and ideas
		Staff role limitations

### Consumer attendance and interest

This theme was on consumer-related factors that affected program delivery.

Staff indicated that adequate consumer attendance determined whether programs were introduced or continued running. When asked about possible deterrents to a new program, Staff 2 said;

*“I think again, it's trying to get a way of engaging clients to come into the centre.”*

Additionally, staff reported that consumer attendance was the primary indicator of program success. This was because consumers did not typically express their feedback verbally due to various impairments.

Consumer interest in prospective programs also determined which programs were introduced in the setting because consumer feedback and ideas were sought prior to implementation. Staff 7 stated this in the interview:

*“Ultimately I have all say about what groups would or wouldn't be delivered. But, at this point in time, because I'm reasonably new, we talk to our participants and ask them what they want.”*

### Availability and restrictions to the use of funding

The availability of funding or the stipulated uses of financial resources, was the second theme on program delivery.

One manager reported that the NDIS was the primary and only way consumer programs were funded, and thus program cost-effectiveness had to be ensured through a staff-to-consumer ratio that was financially justifiable. Staff 7 summarised this by saying:

*“Because of the way NDIS funds, you've got to have a viable number of people in the group against your staffing ratios to make it financially viable."*

In addition, both managers acknowledged that lack of funding was a challenge because service availability was wholly dependent on funding allocated to individual NDIS plans. This funding was generally not allocated to specialised services, such as those offered by allied health, despite the possibility for this under the NDIS. Staff 6 expressed some of these challenges in her statement:

*“If we had had a lot of money, like* [names another centre] *coming in every year, to be able to employ staff and not have to worry about having someone coming, a client coming to be able to pay that staff member, then we could have done a lot more.”*

Finally, NDIS funding covered staff wages and organisational maintenance costs, but not program activity costs, which limited the programs that could be run without charging consumers additional fees for program activities. Staff 7 stated:

*“It* [funding] *covers the support workers and it covers the centre. So anything above that comes out of our bottom line. But NDIS, which is how our customers are funded, are very clear about [sic], they don't pay for food. They don't pay for activity costs themselves.”*

### Organisational structure and practices

The third theme on the issues that affect program delivery was on matters pertaining to the CMO and how it was run.

Facility availability was highlighted as determinant of what programs could be delivered: two staff participants mentioned the importance of having indoor gym equipment at the CMO, as this could promote engagement with physical activity programs and activities. Staff 5 explained it this way:

“*There's no exercise machines here [*sic*]. It would be good if we had one in here. Someone might get on a treadmill, you know. Yeah. And there's not a reason why. I suppose it depends on budget.*”

Additionally, policies and procedures sometimes limited availability of some program activities at the CMO. One manager noted that policies and procedures stipulated that onsite access to gym equipment had to be supervised by specialised staff, who were otherwise unavailable at the CMO. This restricted physical activity program activities. Staff 6 discussed the potential barriers to a new nutrition and physical activity program by saying:

“*Policy and procedures play a big part, like we had the offer of donation of gym equipment, but we got told from Sydney that if you have a gym equipment here* [*sic*] *you've got to have someone qualified on site. So that went out the door*.”

Staff input determined which programs were delivered. Support worker input was requested regarding ideas for prospective programs; however, managers ultimately decided which programs were delivered. Staff 7 explained the process of introducing new programs in the statement:

“*I will usually talk to a staff member and say, "Would you like to be a part of this?" Then we work through the process of what that might look like, what resources they will need, and then we talk through each week, "How did it go," until they're really confident to run the group on their own*.”

Finally, limitations in staff roles and training determined which programs were delivered. One manager mentioned that support workers generally delivered programs, however, they were not trained to provide specialised services. Staff 7 described it thus:

*“You could go to* [states name] *Park and there's all this equipment … … … .. but a general support worker isn't trained to do that, so you actually need a specialist. But there's no funding available for that to work. So that disconnect is quite significant, I think.”*

## Discussion

This study is the first to identify the factors which affect program access and attendance from consumers and staff in a regional CMO. Program access, or attendance was shaped by five themes: consumer financial status, domestic responsibilities and health; program design and delivery; organisational structure and practices; staff attitude, skills and effort; and social connections and stigma experienced by consumers. Furthermore, staff accounts on the elements that impacted program delivery highlighted consumer attendance and interest, availability and restrictions to the use of funding, and organisational structure and practices. An important step to improving the translation of evidence-based lifestyle interventions for people with psychotic illness into CMOs, is the consideration of factors that affect program access and delivery during the process of intervention design and implementation [5, 9, 12, 24]. This greater understanding can enhance efficiency of knowledge translation and promote success of lifestyle interventions [12, 24].

Consumer program access was affected by poor health status, negative medication side-effects, NDIS financial support, domestic responsibilities and motivation levels. Despite lack of comparable research from other CMOs, these challenges commonly occur in people with psychotic illness and other severe mental illness [25]. Previous research identified that illness symptoms can be a barrier to participating in short-term lifestyle intervention research for those with severe mental illness, with antipsychotic treatment further limiting engagement due to side effects [9, 26]. Loss of motivation is a persistent health problem in those with psychotic illness and other severe mental illness that can also be a program access barrier [9, 25]. The overall impact of these health issues can negatively affect financial status, although Australians experiencing psychiatric disability are eligible for service access funding though the NDIS [27]. This funding can be used to purchase services that help maintain independence, health and wellbeing—including assistance for domestic duties [27]. Consumer participants, however, noted that they had to prioritise family and domestic duties over attendance. It is difficult to address all these consumer-related concerns within the context of a CMO, because government collaboration is necessary to manage health and financial issues [28]. However, anticipating and considering these factors during implementation could result in more user-friendly programs for CMO consumers [12, 20].

Consumer engagement with the CMO programs was also influenced by program design and delivery, which comprised the activities offered, convenience of meeting location, transport provision, time of year and time of day that programs were held, consideration of individual needs during delivery, and consumer perceptions about programs. Primary health care research for people with severe mental illness (generally) shows that organised transport provision promotes attendance of appointments, hence should be utilised by CMOs [29]. Other program-related issues pertaining to timing, individual consumer needs, and consumer perceptions, can generally be addressed during implementation without altering fidelity of intervention content [5, 24]. Moreover, program activities in prospective lifestyle interventions can be adapted to suit consumers, if components that lead to efficacy are known [24]. Comprehensive knowledge on elements that contribute to program efficacy is essential for successful design alteration, to ensure efficacious elements are retained during integration of consumer suggestions [24].

Equipment availability and dissemination of information about programs, were the only organisational structure and practices that impacted program access. Translation of lifestyle intervention research into the community could be hampered by equipment discrepancies, if specialised equipment and trained personnel are required [12]. Considering the needs of both the scientific community and real-world practice settings in research and implementation, would enhance intervention usability [12, 24]. Further, adequate dissemination of information regarding CMO programs would minimize any associated service access challenges [30].

The potential effect of staff attitude towards consumers, and relevance of their professional skills during program delivery, were important considerations for consumer program attendance. This was in addition to provision of follow-up by staff, affirming literature that highlights the key role of mental health staff in facilitating or hampering health service attendance [9, 30, 31]. Addressing legitimate consumer concerns, concerning program or service providers, is of great importance in people with psychotic and other severe mental illness, because they face additional societal barriers to access [31]. Stigma associated with mental illness was one of the societal barriers in this study, while value of program social connections was a motivator. Internalised stigma and discrimination inhibit primary health care access and community participation in people with severe mental illness generally [30, 32]. Moreover, adequate social participation is an integral part of improving quality of life for those experiencing psychotic and other severe mental illness, hence should be promoted in programs [9, 32]. In contrast, societal stigma or discrimination is harder to control when delivering programs because public education is necessary [30, 32]. Nevertheless, consulting with consumers and conducting programs in spaces they perceive as non-stigmatizing may alleviate this [32].

Consumer attendance and interest in prospective services were parameters used to determine which programs could be delivered in the CMO, indicating the application of person-centred approach [33]. Clinical research shows that person-centred care enhances consumer empowerment and treatment outcomes [33]. The implication for implementing lifestyle interventions is the involvement of consumers in decision-making within target intervention settings [33].

Program delivery in the CMO was limited by lack of funds to cover program activity costs or employ specialised staff, with the NDIS allotments. Additionally, program cost-effectiveness had to be ensured through a financially justifiable consumer-to-staff ratio, to ensure NDIS funding was sufficient to cover running costs. Programs and services funded under the NDIS are required to minimize impairments that inhibit consumers from performing activities of daily living or engaging in the community [19, 27, 34]. The implication for lifestyle interventions seeking delivery through this avenue is that outcomes ought to demonstrate impact on these parameters [27]. Alternatives to NDIS funding for lifestyle intervention delivery in CMOs, are grants and funding from charitable organisations that support non-for-profits [35]. Literature does not indicate that this has been done previously, but it could be trialled in future to create a case for continued funding.

Organisational structure and practices that influenced program delivery, including the availability of facilities, policies and procedures, staff input and ideas, and the role limitations of staff, generally require consideration prior to lifestyle intervention implementation [12]. Facilities at a CMO may limit lifestyle intervention delivery, if specialised equipment or spaces are required [36]. In addition, all proposed programs would need to demonstrate adherence to policies and procedures especially with regard to work health and safety, as breaches can lead to harm of persons and costly fines [37]. Finally, incorporating ideas from CMO staff into the design of simple lifestyle interventions that can be delivered by a range of professionals, may promote program success in this context [12]. This will ensure that programs have the intended impact on consumers [12, 24].

### Study strengths and limitations

Although the present study was characterised by a relatively small sample size (*n* = 13), information provided on the factors which affect program access, mirrors some previously reported challenges in people with psychotic and other severe mental illness [9, 25, 32]. Findings may therefore provide a template for issues which can be evaluated by other CMOs when delivering programs for this population [9, 12, 25, 32]. However, information on elements to consider during program delivery may differ depending on context, and caution should be observed prior to application of current findings [12]. In light of the resource and time limitations confronted, adoption of semi-structured interviews rather than unstructured interviews promoted research efficiency; nonetheless, unstructured interviews may have produced richer data especially with regard to the personal lives of consumers and how they spend their time whilst attending programs [38].

## Conclusion

This study identified the factors that affect program access and delivery in a CMO delivering services to people with mental illness. Consideration of the issues cited by consumers and staff led to the notion that various strategies could be applied to address challenges associated with the programs delivered, the organisation structure, staff involved, consumer social issues and funding available for programs [9, 24]. Although factors pertinent to the personal lives of consumers’ presented as problematic to address, anticipating and planning for these complexities may alleviate some of the associated effects [39]. Translation of evidence-based lifestyle interventions in CMOs, for people with psychotic illness, necessitates the consideration of issues that affect program access and delivery during the process of intervention design and implementation [5, 9, 12, 24]. This will enhance efficiency of knowledge translation and promote success of the lifestyle interventions [12, 24].

## Supplementary information

**Additional file 1.** Interview Questions for Patricipants.

## Data Availability

The data generated and analysed in the current study are not publicly available, in order to protect the confidentiality of the study site and participants; however, further data to support the current findings can be provided by the corresponding author upon reasonable request.

## Abbreviations

BMI: Body mass index
CMOs: Community managed organisations
NDIS: National Disability Insurance Scheme
NDIA: National Disability Insurance Agency
i-PARIHS: Integrated Promoting Action on Research Implementation in Health Systems”
