# Supplementary material for: Factors to consider during the implementation of nutrition and physical activity trials for people with psychotic illness into an Australian community setting
Source: BMC Health Serv Res. 2020 Aug 12;20:743. doi: 10.1186/s12913-020-05629-0 (PMC7425062; doi:10.1186/s12913-020-05629-0)
Supplement: Supplementary file 1 — Additional file 1. Interview Questions for Patricipants. [file 12913_2020_5629_MOESM1_ESM.docx]

Interview Questions for the Area Leader

1. What are some of the services and programs offered to clients at this branch of (Organisation named)?
2. What is the process of selecting programs that are delivered to clients?
3. Do you have a system in place that assesses the relevance of current programs to client needs? **If applicable*
   1. How do you do this?
4. Do you evaluate the success of programs? **If applicable*

a. How do you do this?

1. Are there factors that make a program sustainable over time? **If applicable*

a. What are these factors?

b. What kind of programs have been discontinued?

1. Are there regulations that new programs have to abide by?

a. What are these regulations?

1. How are programs funded?
   1. Are there challenges associated with obtaining this funding? **If applicable*
   2. What are these challenges?
   3. What is the process of navigating through these challenges?
2. Are there organisational resources that are available for programs?

a. What are these resources?

1. How do you initiate new programs?
2. Are staff trained to deliver new programs? **If applicable*

a. How is this done?

1. Do you think there is a current need for an evidence-based healthy living program which focuses on good nutrition and adequate physical activity? **Prompt*
   1. What are some of the reasons behind your answer?

*If participant does not see the need for a healthy living program, then end the interview here*

12. What are the barriers to the implementation of the healthy living program?

a. What are the enablers to the implementation of the healthy living program?

13. Which individuals should be involved in the implementation of the healthy living program?

14. Are there some incentives that can be put in place to promote interest and engagement with the healthy living program by staff/ clients?

a. What are these incentives?

Interview Questions for Adults (18-64 years) with Psychosis or Other Severe Mental Illness

1. What programs do you attend at (Organisation named)?
   1. Do you choose these programs? * *If applicable* How do you choose these programs? *(Prompt if necessary for information related to cost, content and duration of programs, program days and time)*
   2. How do you find out about programs?
   3. What do you enjoy about the programs that you attend?
   4. What do you not enjoy about the programs you attend?
   5. Why do you continue attending certain programs?

f. Why do you discontinue certain programs?

1. What do you like about how staff run programs?
2. What do you not like about how staff run programs?
3. Are there things that make it difficult to attend programs?

a. What are these things? * *If applicable*

b. Does your health affect participation or your attendance of programs? **if not mentioned*

c. If so how?

1. Would you like to be supported by staff if it’s harder to attend or participate in programs because of your health? * *If applicable*

e. If so how?

1. Do you think you need a new healthy living program at (Organisation named) which promotes good nutrition and adequate physical activity to help you maintain your health? **Prompt*
   1. Can you please explain some of the reasons behind your answer?

*If participants do not see the need for a healthy living program, then end the interview here. If they, however, see the need for a healthy living program, then proceed*

1. Would you like to receive education as part of the healthy living program? * *If applicable* a. What are some of the topics you would like covered?
2. How often would you consider attending the healthy living program? **Prompt*
   1. What are some of the reasons behind your answer?
3. Who would you like to see deliver the healthy living program? **Prompt*
   1. What are the reasons behind your answer?
4. Do you have some personal goals you would like to achieve if you attended the healthy living program? * *If applicable*

a. What are these personal goals?

1. Would program cost influence your decision to attend the healthy living program? **Prompt*
   1. Please give me some reasons behind your answer
2. How would you feel about attending the healthy living program with a friend or family member if you chose to attend this program? **If applicable*
   1. Please give me some reasons behind your answer

Interview Questions for Support Workers, Primary Recovery Support Worker and Site Coordinator

*Participants*

1. Are you involved in running or developing programs delivered at (Organisation named)? **If applicable*

a. What role/s do you play in the programs?

1. Do you know how the programs you are involved with were developed? **If applicable*
   1. Please tell me how?
2. Do you receive support prior to running new programs?
3. What kind of support do you find helpful?

d. What kind of support do you not find helpful?

1. How would you define evidence-based practice? **If no answer, the following definition will be provided.*

***Evidence-based practice is the diligent and judicious application of the best evidence from research, the clinical environment and patient values to guide healthcare in patients****.*

- 1. Is evidence-based practice applied when delivering programs? **If applicable*
  2. How is this done? ** Prompt – Is there a process or guideline that (Organisation named) follows when implementing a program?*

1. What affects your motivation in your involvement with client programs?
2. Can you usually gauge if clients are enjoying programs? **If applicable*
3. How do you do this?
4. Are there factors that are associated with the clients’ continued participation or engagement with programs? **If applicable*

a. Please explain some of these factors?

1. How do you define program success?
   1. What features in a program make it successful?
2. Do you see the need for a new healthy living program focusing on good nutrition and adequate physical activity at (Organisation named)? ** Prompt*
   1. Please give me the reasons behind your answer? **If applicable*

*If participants do not see the need for a healthy living program, then end the interview here*

1. What would you like to see the healthy living program achieve?
2. How do you think clients would benefit from the healthy living program?
3. Are there some things in the workplace that would make it difficult to have the healthy living program? **If applicable*
   1. Please tell me about these things
   2. Tell me about any workplace changes that might be needed to make it possible to have the healthy living program?
4. Would you need any support in the workplace if you were to be involved with the healthy living program? **If applicable*

a. What kind of support do you anticipate that you would need?
